# Multilocus Genotyping of *Pneumocystis jirovecii* from Deceased Cuban AIDS Patients Using Formalin-Fixed and Paraffin-Embedded Tissues

**DOI:** 10.3390/jof7121042

**Published:** 2021-12-05

**Authors:** Vicente Friaza, Yaxsier de Armas, Virginia Capó, Rubén Morilla, Arturo Plascencia-Hernández, Héctor R. Pérez-Gómez, Enrique Iglesias, Luis Fonte, Carmen de la Horra, Enrique J. Calderón

**Affiliations:** 1 Instituto de Biomedicina de Sevilla, Hospital Universitario Virgen del Rocío/Consejo Superior de Investigaciones Científicas/Universidad de Sevilla, 41013 Seville, Spain; vfriaza-ibis@us.es (V.F.); rmorilla2@us.es (R.M.); sandube@cica.es (E.J.C.); 2 Centro de Investigación Biomédica en Red de Epidemiología y Salud Pública (CIBERESP), 28029 Madrid, Spain; 3 Department of Clinical Microbiology Diagnostic, Hospital Center of Institute of Tropical Medicine “Pedro Kourí”, 11400 Havana, Cuba; Yaxsier@ipk.sld.cu; 4 Pathology Department, Hospital Center of Institute of Tropical Medicine “Pedro Kourí”, 11400 Havana, Cuba; capovir@infomed.sld.cu; 5 Centro Universitario de Ciencias para la Salud, Universidad de Guadalajara, 44100 Guadalajara, Mexico; aplascenciah@yahoo.com.mx (A.P.-H.); hectorraul.perez@cucs.udg.mx (H.R.P.-G.); 6 Centro de Ingeniería Genética y Biotecnología, Departamento de Vacunas, 10600 Havana, Cuba; enrique.iglesias@cigb.edu.cu; 7 Parasitology Department, Institute of Tropical Medicine “Pedro Kourí”, 11400 Havana, Cuba; luisfonte@infomed.sld.cu

**Keywords:** pneumocystis, genotype, epidemiology, autopsied lungs

## Abstract

The results of the genotypic characterization of *Pneumocystis jirovecii* are described in lung tissue samples from 41 Cubans who died of AIDS with pneumocystosis between 1995 and 2008. Histological sections of the lung preserved as formalin-fixed and paraffin-embedded tissue were examined. PCR amplification and nucleotide sequencing of the two mitochondrial genes (large and small) of the pathogen allowed verification of a predominance of genotype 3 (85T/248C) of the large mitochondrial gene and genotype 3 (160A/196T) of the small mitochondrial gene over a period of 14 years (1995–2008). These results suggest that the 85T/248C//160A/196T genotype circulates with the highest frequency (81.3%) among AIDS patients in Cuba. Multilocus analysis indicates a limited circulation of pathogen genotypes on the island with the existence of a clonal genotype with an epidemic structure. Furthermore, it appears that circulating strains of *P. jirovecii* have not developed mutations related to sulfonamide resistance. Taken together, the data in this study revealed important elements about pneumocystosis in Cuban patients dying of AIDS and the usefulness of formalin-fixed and paraffin-embedded samples to carry out molecular epidemiology studies of *P. jirovecii*.

## 1. Introduction

*Pneumocystis jirovecii* pneumonia (PcP) is considered one of the most common opportunistic diseases in individuals infected with human immunodeficiency virus (HIV). Despite the use of antiretroviral therapy (ART) and the chemoprophylaxis recommended for its control, PcP continues to have a negative impact on the health of patients with AIDS in many countries. Even today, it is the most prevalent opportunistic infection in AIDS patients, and it is also high among subjects with non-HIV-related immunosuppression [1,2].

The lack of a microbiological culture medium for *P. jirovecii* is an important obstacle in fully understanding the epidemiology of PcP, as well as its biology and susceptibility/resistance pattern to drugs to improve treatment [1,3]. Thus, to overcome these drawbacks, molecular tools are being used for the detection and characterization of this pathogen. Among the molecular targets used to study the microorganism are the genes that encode the large and small subunit of mitochondrial ribosomal RNA (*mt LSU rRNA* and mt *SSU rRNA*, respectively) and the gene that encodes dihydropteroate synthetase (DHPS) [4,5]. In this last gene, some point mutations have been related to resistance to drugs indicated for PcP treatment and prevention based on homologies to other microorganisms and epidemiological data [6].

The diagnosis of PcP is confirmed by microscopic observation of any life stage of the pathogen in respiratory samples. In this sense, the samples obtained by bronchoscopy provide a better score of positive detection. However, collecting this type of sample requires invasive medical management, therefore this procedure is not usually performed in many countries to identify the pathogen [1,7]. In view of this limitation, formalin-fixed and paraffin-embedded tissues (FFPE) could be alternative sources of samples for molecular studies [8,9]. However, the number of reports favoring the use of FFPE in *P. jirovecii* studies is still scarce, and, in addition, they are more directed toward identification than to characterization of the microorganism [10,11,12,13]. 

The purpose of the present work was to: (i) confirm genotypically *P. jirovecii* samples in FFPE tissues of Cuban patients who died of AIDS using the *mt LSU rRNA* and *mt SSU rRNA* genes, (ii) identify *P. jirovecii* strains with potential resistance to sulfonamides by studying the *DHPS* gene, and (iii) evaluate the feasibility of two polymerase chain reactions for the molecular characterization of *P. jirovecii* in formalin-fixed and paraffin-embedded tissue samples.

## 2. Materials and Methods

The study included autopsies performed in the Department of Pathology of the Institute of Tropical Medicine Pedro Kouri (IPK) in 514 patients who died of AIDS during the period from January 1995 to May 2008. Histological sections were prepared from their lung tissue in FFPE. Histopathological diagnosis of infection by *P. jirovecii* was made by light microscopy with hematoxylin and eosin and Gomori methenamine silver staining. This procedure is considered the gold standard technique to identify *P. jirovecii* by visualizing the pathogen in the analyzed tissue. A total of 41 lung tissue samples were selected from 41 decedents who died of AIDS-related PcP (Table 1).

In each of the cases, a sample of approximately 1.0 g of deep lung tissue was taken using sterile equipment, placed in sterile receptacles, and fixed in buffered formalin for 24h under aseptic conditions. Only one lung was processed in a single day. Five-micrometer serial sections (0.3 µm) from FFPE tissue samples were used for DNA extraction that was performed using the commercial Qiagen NucleoSpin^®^ Tissue Kit (Hilden, Germany) following the manufacturer’s instructions [14].

Genotypic characterization of *P. jirovecii* strains was carried out using the genes *mt LSU rRNA* (260 bp fragment) and *mt SSU rRNA* (308 bp fragment), as described by Wakefield in 1998, and Hunter and Wakefield in 1996 [15,16]. After amplification of the fragments, nucleotide sequencing was performed with the commercial package ABI Prism dRhodamine Terminator Cycle Sequencing Ready Reaction Kit (PE Applied Biosystems, Foster City, CA, USA) and polymorphism was determined at positions 85 and 248 for the *mt LSU rRNA* gene, and 160 and 196 for the *mt SSU rRNA* gene [14,15,16].

To identify *P. jirovecii* strains with mutations related to resistance to sulfonamides, a restriction enzyme analysis of the gene encoding dihydropteroate synthetase (DHPS) was used [17]. The length of the polymorphism in the *DHPS* gene was detected at nucleotide positions 55 and 57 by enzymatic restriction with the HaeIII and AccI enzymes, respectively. Briefly, 50 mL of the PCR products were digested with AccI and HaeIII (1 U/mL, final concentration) separately, at 37 °C for one hour. The 50 µL of the PCR-restriction fragment-length polymorphism (RFLP) was divided into 3 aliquots. One was used to confirm the presence of a 370 bp fragment from the *DHPS* gene. The second and third aliquots were used to identify the presence of wild-type versus mutations in codons 55 and 57 by RFLP with AccI and HaeIII (Roche Diagnostics), respectively. When a wild-type sample containing no mutation in codon 55 is digested with AccI, two DNA bands appear at 229 and 141 bp. When the mutation is present, only one uncut band appears at 370 bp. Similarly, with HaeIII, two bands appear at 221 and 149 bp in wild-type samples without mutation in codon 57, and only one uncut 370 bp band appears if mutation is present [18].

Evaluation of the feasibility of two other PCRs for the characterization of *P. jirovecii* in FFPE was carried out by amplifying a 136 bp fragment of the gene that encodes the *mt LSU rRNA* gene as described by Wakefield et al. [4]. For the same purpose, the 186-bp sequence of the gene encoding the DHPS of *P. jirovecii* was used for RFLP analysis [19]. In this case, when the mutation is present, a 186 bp band appears. Similarly, after RFLP, 2 bands at 148 and 38 with AccI, and 140 bp and 46 bp with HaeIII in wild-type samples appear [19]. 

To determine discriminatory power (ability of a typing method to differentiate between two unrelated samples), the Simpson index of diversity (S) was calculated [20]. The S-index was determined for each locus (*mt LSU rRNA* and *mt SSU rRNA*):

$$S-index=1-\left[1÷N\left(N-1\right)\overset{S}{\underset{j=1}{\sum }}nj\left(n1-1\right)\right]$$

*N*: number of isolates in the sample population. *S*: total number of types described. *nj*: number of isolates belonging to the *j*th type.

All medical records (MR) of the deceased patients involved in the study were reviewed. Sociodemographic, laboratory and clinical management of patient variables were collected. Among sociodemographic variables, the following were analyzed: age, sex, skin color, occupation of the deceased (technical, professional, worker, student, housewife, inmate, retired) and current place of residence (province of residence recorded upon admission to the hospital). Regarding the variables of clinical management of the patient, the following were collected: year of HIV diagnosis, year of death, use of antiretroviral treatment (yes/no), exposure to sulfa drugs in the three months prior to death (yes/no), treatment with sulfa drugs upon admission (yes/no), and diseases or coinfections. Concomitant diseases were diagnosed by histopathological and microbiological analysis. Only one clinical laboratory variable was collected from MR, the number of CD4 + T lymphocytes (cells/µL) obtained a month before the death of each patient.

The association of the genotypes obtained for the *mt LSU*, *mt SSU rRNA* genes and their combination (*mt LSU/mt SSU rRNA*) with age, sex, antiretroviral treatment, sulfonamide treatment on admission and the number of CD4 + T lymphocytes was determined using the Chi square test. A *p*-value < 0.05 was considered statistically significant.

All participants or their relatives completed the written informed consent and, according to our hospital’s regulations, the procedure for requesting and authorizing research studies was completed.

## 3. Results 

The 260 bp fragment of the *P. jirovecii mt LSU rRNA* gene was amplified in 26 samples (63.4%) from the 41 paraffin blocks analyzed. Regarding the genotype distribution of mt LSU, there was a predominance of genotype 3 (23 samples (88.5%)) during the 14-year duration of the study. At the same time, two sequenced samples corresponded to genotype 1 (7.7%), and the presence of genotype 2 was identified in a single sample (3.8%), which corresponded to a mixed infection of genotypes 2 and 3.

Furthermore, of the 26 samples positive for the *mt LSU rRNA* gene, 16 (61.5%) were also amplified and sequenced for the *mt SSU rRNA* gene, which corresponds to 39.0% of the 41 paraffin blocks analyzed. We found that 15 (93.8%) of these samples corresponded to genotype 3 (160A/196T), a genotype whose sequence was reported to the Gene Bank with accession number (HQ228547) [14]. The remaining sample corresponded to genotype 2 (160A/196G) [21]. 

Taken together, multilocus analysis comprising the results of the *mt LSU* and *mt SSU rRNA* genes identified the genotype (85T/248C//160A/196T) in 13/16 (81.3%) as predominant in the 16 samples that could be analyzed for both markers. 

Using primers to amplify the DHPS-encoded gene, a 370 bp fragment was obtained in two samples (4.9%) of the 41 paraffin blocks analyzed in this study. In both cases, the absence of point mutations was observed in this gene. To evaluate whether selection of smaller fragments could increase the rate of positive amplification, a 136 bp fragment from the *mt LSU rRNA* gene and another 186 bp fragment from the gene encoding DHPS were analyzed. In surprising contrast, the 136 bp fragment of the mt *LSU rRNA* gene was amplified in all 41 FFEP samples analyzed. Sequencing of this fragment was achieved in 35/41 (85.4%) of the amplified products. Analysis of the polymorphism at position 85 showed that 32 (91.4%) samples had T at that position, two samples (5.7%) C, and in one sample (2.9%) A and T were simultaneously identified in that position. The last sample corresponded to a mixed infection. It is interesting that there was 100% coincidence with the previous 26 samples which amplified the fragment of 260 bp. On the other hand, the 186 bp fragment of the *DHPS* gene was amplified in seven samples (17.1%) of the 41 paraffin blocks analyzed. In the seven samples (two amplified by 370 bp PCR and five additional ones), the absence of point mutations was demonstrated. The patterns corresponding to the wild genotype were identified when applying the enzymatic restriction using AccI and HaeIII.

The *mt LSU* locus exhibited the highest S-index for genotypes (S = 0.2185), for *mt SSU* the S-index is 0.125.

The mortality rate due to PcP calculated for the period 1995-2008 in patients with HIV/AIDS was 7.98% (41/514). It is important to note that 29/41 (70.7%) of the patients included in this work died before the ART prescription was available in Cuba (May 2000). The remaining 12 patients (29.3%) received some antiretroviral treatment while still alive; of these, five (41.7%) were after May 2000. On the other hand, 78.0% of the cases were treated with sulfa drugs at admission and 29.3% had previously been exposed to sulfa drugs in the last three months. In none of these cases, the *DHPS* gene was amplified (Table 1). 

The mean number of CD4 + T lymphocytes in the patients was 90.8 ± 12.9 cells/µL (range 10–308), with 62.6% severely immunosuppressed (values less than 100 cells/µL). 

The highest frequency of HIV infection diagnosis among the deceased in the study was made in 1998 (12.2%), the same way that it coincided with the highest frequency of cases of deceased per year, 14.8%. The comparisons in this study did not reach a statistically significant association between the genotypes obtained from the sequencing of the *mt LSU rRNA*, *mt SSU rRNA* genes and their combination with the variables age, sex, antiretroviral treatment, treatment with sulfonamides at admission or the number of CD4 + T lymphocytes.

## 4. Discussion 

Most of the genotypic characterization studies of *P. jirovecii* were made using respiratory samples, such as bronchoalveolar lavages, spontaneous and induced sputum and oral lavage from patients with suspected PcP [1,3,4]. Few studies pursue this purpose in FFPE [10,11,12,13]. In this type of sample, DNA is usually fragmented, and minimal amounts of genetic material can be found intact. Thus, the real possibilities of a successful PCR are limited. Furthermore, the time of exposure to heat and formalin and the temperature for the preservation of tissue samples are important variables to consider [9,14]. Therefore, it was not unexpected that only 63.4% of the analyzed samples were positive for amplification of the 260 bp fragment of the *mt LSU* gene. This finding is consistent with reports from other authors who have used this type of material to perform genetic studies [8,9,13,14]. To our knowledge, this study is the first attempt to genotype *P. jirovecii DHPS* in patients with AIDS-related PcP from FFPE tissue and confirm our previous data on mitochondrial genes using a larger number of samples [14]. 

Of the five possible combinations that have been described for the *mt LSU rRNA* gene, three genotypes were identified: genotype 3 in almost all samples, genotype 1 in very few samples, and coinfection with 3 and 2 only in a single sample. These results coincide with those described in 14 HIV patients with PcP in Zimbabwe, with a prevalence of 57.0% (genotype 3) [22]. Consistent with this, Le Gal and his colleagues described 44.4% of genotype 3 in nine AIDS patients from French Guyana [23]. However, there are differences with other regions of the world. For example, in cities such as Seville, Lisbon, London, and Sydney, genotype 1 is predominant and is the most common genotype reported in Europe [24,25,26]. Differences in prevalence of *mt LSU rRNA* genotypes depend on factors that are inherent to specific conditions in an area or region. In fact, climatic and geographical characteristics can influence the distribution, circulation, and transmission of different genotypes of *P. jirovecii*. It is important to highlight that regions with similar climatic conditions have a trend toward a predominance of the same genotype [14,23,26]. 

On the other hand, genotype 2 was only found in one mixed infection with genotype 3 (3.8%). A similar frequency was found by Montes-Cano et al. in Spain in 2004 [27]. However, it differs from that reported by Gupta et al. in India, and Beard et al. in the USA (6.0% and 10.2%, respectively) [28,29]. In a previous report by our group, no cases with genotype 2 were identified in Cuba, in contrast to 50% identified in France [14]. 

One caveat of our analysis is that Sanger sequencing-based methods are less sensitive than other methods. They detect multiple genotypes in only about 30% of the samples. For this reason, discrepancies have been obtained when other molecular tools were used for genotyping [30]. For example, Hauser et al. selected four different regions of the genetic material of the fungus and used the SSCP technique and found about 77% of mixed infections in their samples [31]. Similarly, 70% of multiple genotypes obtained by STR have been described [30]. Unfortunately, those methods have not been tested on FFPE. However, recovery of unique genotypes suggests the hypothesis of transmission among these patients. The presence of multiple genotypes in the same patient can be related to three important aspects. First, coinfection of different strains of *P. jirovecii*; second, mitochondrial DNA can undergo more recombination events and acquire more mutations over time than nuclear DNA; and third, continuous exposure throughout life, together with the active multiplication of a subset of strains during immunosuppression [32]. Previous studies revealed that 80% of samples harboring mixed sequences are obtained when nuclear gene targets were used, and this proportion increased to 92% when a mitochondrial DNA target was added to the ultra-deep pyrosequencing analysis [32].

Another of the genes involved in this study for the genotypic characterization of *P. jirovecii* samples was the *mt SSU rRNA* gene. For this gene, only 39.0% of the samples were amplified, in contrast to the 63.4% value obtained with the *mt LSU rRNA* gene. This shows that despite both genes being organized in multiple copies and generating similar amplification products (260 bp versus 308 bp), the PCR that uses the *mt LSU rRNA* gene as a target was 1.63 times more sensitive than its mitochondrial counterpart. Several previous reports agree with this result when they evaluated different genes of the pathogen in patients with PcP [33,34,35].

Several authors consider that the *mt SSU rRNA* gene has less discrimination power than the *mt LSU rRNA* gene, since only two positions analyzed (160 and 196) in a 338 bp portion of the gene present a certain degree of polymorphism [14,36,37]. According to Hunter et al. [16], four possible nucleotide combinations can be obtained (in 160, the A and C, while in 196, it presents the T and G), of which, to date, three genotypes have been described in the literature: genotype 1 (160C/196T), genotype 2 (160A/196G), genotype 3 (160A/196T) [14,16]. The latter was mainly found in the samples analyzed in the present study (93.8%). This genotype was previously known to be the most prevalent in France, Spain, and Cuba [14]. The results of this work suggest the need to incorporate this gene into the multilocus analyses carried out and to develop other studies that investigate the possible role of this gene as a geographic marker. In addition, it is necessary to provide evidence on associations of the *mt SSU rRNA* gene genotypes with the severity of the disease, as well as with the clinical and epidemiological data of the patient. 

By analyzing both genes simultaneously (multilocus analysis), interesting findings were revealed. The investigation demonstrated a maintained prevalence over time of the 85T/248C//160A/196T combination (13/16 samples sequenced by the *mt SSU rRNA* gene) in the samples analyzed in the present work. Although the number of cases evaluated in this work is relatively small, the results suggest two important conclusions: (a) there was a selection for the 85T/248C//160A/196T genotype among Cuban HIV seropositive patients coinfected with HIV-1/*P. jirovecii* at least during the study period and (b) active transmission of this genotype among severely ill patients dying of AIDS. On the other hand, the low genetic diversity detected in the studied samples suggests a hypothesis about the narrow circulation of this combination of mitochondrial genotypes on an island, a situation that contrasts with what happens on continents, where a mixture of genotypes is generally observed [14,24,25,26]. This information could clarify important aspects of the epidemiology of *P. jirovecii* and allow specific decision-making strategies for the management of this infection. 

The third gene evaluated in this work was the one that encodes DHPS, a key enzyme in the metabolism of folic acid and involved in resistance to sulfonamides. Only two samples of the 41 paraffin-embedded tissues were positive using PCR that amplifies a 370 bp fragment, which represents 4.9%. Using a PCR that amplifies a 750 bp fragment in FFPE of the same gene, Robberts et al. did not obtain any amplification in 12 samples obtained from patients with AIDS from a South African hospital [34]. The previous result indicates that it is not easy to amplify fragments of the DHPS gene in FFPE. Therefore, more studies are necessary to evaluate a larger number of respiratory samples from patients suspected of PcP to truly know the impact of the presence/absence of resistance strains of *P. jirovecii* to sulfonamides in Cuban patients. 

In this study, the use of a smaller molecular size fragment (186 bp) of the *DHPS* gene increased the detection frequency more than three times in FFPE. This shows that not only does the repetitive sequence in the genome guarantee the success of PCR, but it is also important that the size of the sequence to be amplified does not exceed 300 bp due to the degradation of DNA in FFPE [34,35,38,39]. Very few studies on FFPE in the literature address this issue [8,13,14]. On the other hand, all amplifications of the *DHPS* gene obtained in this series were wild-type genotypes, indicating the absence of point mutations. This study, despite the small number of samples, is the first study in Cuba to address the situation of resistance to sulfonamides in *P. jirovecii* in adults with HIV. Furthermore, it suggests that so far there is no resistance to this drug in the country despite its use as chemoprophylaxis in patients who died of AIDS with PcP, which contrasts with what happens in developed countries where high resistance rates have been described [6].

The other small fragment for PCR amplification evaluated in this study to achieve the characterization of *P. jirovecii* in FFPE samples is a 136 bp fragment of the *mt LSU rRNA* gene. In the past, this PCR was used in the molecular detection of *P. jirovecii* in samples from patients with lung neoplasms [11]. It is also interesting that all samples analyzed (41 in total) were positive when using this amplification system, in contrast to the 63.4% obtained with the 260 bp PCR. However, six of those 41 samples (14.6%) could not be sequenced. This phenomenon has been observed by several authors who suggested that when the amount of microorganism present in the sample is close to the detection threshold of the technique, there is a risk of failure when sequencing the product [34,35]. In any case, after sequencing the products of PCR-136 bp and PCR-260 bp, there was a total coincidence of the nucleotide at position 85. In the remaining nine samples that were amplified only in PCR-136 bp, T was identified at position 85 of the *mt LSU rRNA* gene. Unfortunately, due to the size of the amplified fragment (136 bp), the previous results obtained with PCR-260 bp at position 248 of the said gene could not be corroborated. However, some articles describe that T is usually the nucleotide base most frequently found at that position [12,13,14]. 

The present work has several limitations: (a) it was a study carried out in a single hospital center, although with national reach (IPK is the reference center for the study of HIV/AIDS in Cuba); (b) the homogeneity of the results in the genes analyzed that separately have a low discrimination power (Hunter–Gaston discriminatory index < 0.3) [20] and (c) the retrospective analysis of this study may influence the results. Other prospective investigations in the future involving different population groups might provide interesting associations between genetic and clinical/epidemiological variables. Regarding the use of FFPE samples, some limitations related to ensuring higher PCR efficiency in FFPE samples were previously discussed. Other factors must also be taken into account, for example, the length and temperature of tissue fixation, chemical reagents used for fixation, temperature, and humidity of the place where FFPE samples were stored, as well as the quantity and thickness of cuts to be used in PCR [8,9,10,14,40]. Therefore, all of these variables should be controlled to increase the frequency of detection of pathogens of medical importance in FFPE samples.

## 5. Conclusions

The present work is a first approach to evaluate the usefulness of FFPE samples for the molecular characterization of *P. jirovecii* and the first to describe the evolution of *Pneumocystis* genotypes in HIV patients over a long period in Cuba. The amplification of two mitochondrial genes (*mt LSU* and *mt SSU rRNA*) demonstrated the feasibility of this type of sample to carry out molecular epidemiological studies. Furthermore, our data confirm that the use of primers that generate fragments of low molecular size and the selection of repetitive regions in the genome of the microorganism are crucial elements to achieve successful amplifications. Overall, the findings of this work suggest a predominant circulation of the 85T/248C genotype of *P. jirovecii*//160A/196T genotype among Cuban HIV seropositive patients who died of AIDS-related PcP. 

## Figures and Tables

**Table 1:** Demographic and clinical data of the patients included in the study.

Characteristics	Deceased Patients N = 41 (%)
**Age in years** 20–39 40–59 ≥60	35.4 ± 1.6 years with a range of 20–60 years 30 (73.2 %) 9 (21.9%) 2 (4.9%)
**Sex** Male/Female	33 (80.5%)/8 (19.5%)
**Skin** White/Others	30 (73.2%)/11 (26.8%)
**Deceased according to antiretroviral therapy prescription** Before/After	29 (70.7%)/12 (29.3%)
**Sulfa drugs treatment** Yes/No	32 (78%)/9 (22%)
**Previous exposure to sulfa drugs** Yes/No	12 (29.3%)/29 (70.7%)
**Number of CD4 + T lymphocytes** <50 cells/µL 50–100 cells/µL 101–200 cells/µL >200 cells/µL No data	13 (40.6%) 9 (22%) 8 (25%) 2 (6.3%) 9 (22%)
